# Giant Arachnoid Granulations: A Systematic Literature Review

**DOI:** 10.3390/ijms241613014

**Published:** 2023-08-21

**Authors:** Rupal I. Mehta, Rashi I. Mehta

**Affiliations:** 1 Department of Pathology, Rush University Medical Center, Chicago, IL 60612, USA; 2 Rush Alzheimer’s Disease Center, Rush University Medical Center, Chicago, IL 60612, USA; 3 Department of Neuroradiology, Rockefeller Neuroscience Institute, West Virginia University, Morgantown, WV 26506, USA; rashi.mehta@hsc.wvu.edu; 4 Department of Neuroscience, Rockefeller Neuroscience Institute, West Virginia University, Morgantown, WV 26506, USA

**Keywords:** GAG, giant arachnoid granulation

## Abstract

Giant arachnoid granulations (GAGs) are minimally investigated. Here, we systematically review the available data in published reports to better understand their etiologies, nomenclature, and clinical significance. In the literature, 195 GAGs have been documented in 169 persons of varied ages (range, 0.33 to 91 years; mean, 43 ± 20 years; 54% female). Prior reports depict intrasinus (i.e., dural venous sinus, DVS) (84%), extrasinus (i.e., diploic or calvarial) (15%), and mixed (1%) GAG types that exhibit pedunculated, sessile, or vermiform morphologies. GAG size ranged from 0.4 to 6 cm in maximum dimension (mean, 1.9 ± 1.1 cm) and encompassed symptomatic or non-symptomatic enlarged arachnoid granulations (≥1 cm) as well as symptomatic subcentimeter arachnoid granulations. A significant difference was identified in mean GAG size between sex (females, 1.78 cm; males, 3.39 cm; *p* < 0.05). The signs and symptoms associated with GAGs varied and include headache (19%), sensory change(s) (11%), and intracranial hypertension (2%), among diverse and potentially serious sequelae. Notably, brain herniation was present within 38 GAGs (22%). Among treated individuals, subsets were managed medically (19 persons, 11%), surgically (15 persons, 9%), and/or by endovascular DVS stenting (7 persons, 4%). Histologic workup of 53 (27%) GAG cases depicted internal inflammation (3%), cystic change consistent with fluid accumulation (2%), venous thrombosis (1%), hemorrhage (1%), meningothelial hyperplasia (1%), lymphatic vascular proliferation (1%), and lymphatic vessel obliteration (1%). This review emphasizes heterogeneity in GAG subtypes, morphology, composite, location, symptomatology, and imaging presentations. Additional systematic investigations are needed to better elucidate the pathobiology, clinical effects, and optimal diagnostic and management strategies for enlarged and symptomatic arachnoid granulation subtypes, as different strategies and size thresholds are likely applicable for medical, interventional, and/or surgical treatment of these structures in distinct brain locations.

## 1. Introduction

Arachnoid granulations (AGs) are macroscopically visible arachnoid tissue outpouchings that protrude into bone, dura, and/or dural venous sinuses (DVSs) [1]. Historically, they have been defined by their juxtaposition and drainage into the superior sagittal sinus (SSS) and other DVSs. AGs primarily consist of collagen, immune cells, and cerebrospinal fluid (CSF)-filled spaces situated at brain borders [1]. These structures were described in 1543 by Vesalius [2] and were further characterized by Willis in 1664, Littre in 1684, Collins in 1685, Mery in 1701, and Pacchioni in 1705 [3,4,5]. However, they were only recently systematically characterized through detailed radiologic–pathologic investigation incorporating comprehensive analyses with cellular and molecular markers, thus enhancing knowledge of their anatomy and potential functions [1].

Typically, AGs are asymptomatic structures that abut dural tissues and measure only a few millimeters in diameter [1,6], but they occasionally enlarge to form so-called giant arachnoid granulations (GAGs) that may also associate with bone marrow spaces and, rarely, scalp dermal tissue. These structures may also cause clinical symptoms and/or nodular DVS filling defects on venography that manifest secondary to flow aberrations, DVS expansion, venous stenosis, and/or other suspected pathologies. GAGs have increasingly been reported in recent years with several cases involving the DVS and others found in extrasinus calvarial or diploic locations. To better understand their pathophysiology and structure, we systematically reviewed the published literature and present an overview of available data pertaining to GAG presentation, histology, imaging characteristics, treatment courses, and outcomes.

## 2. Methods

**Figure 1:**
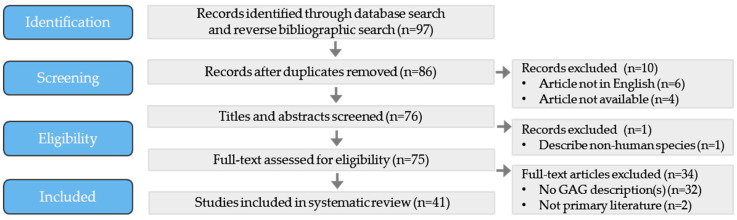
PRISMA flow diagram.

With the understanding that GAGs represent enlarged AGs, published cases of GAGs were searched using PubMed and Scopus to identify original articles and reviews reported in the English language, up to and including July 2023, using a Boolean search strategy incorporating the following terms: ‘Giant’ OR ‘Large’ AND ‘Arachnoid granulation’ OR ‘Pacchionian granulation’ OR ‘Pacchionian body’. Given the paucity of reports meeting this search strategy, the approach was supplemented by a reverse bibliographic search of published cases, series, and reviews. The literature was reviewed by both investigators, guided by the standards of the Preferred Reporting Items for Systematic Review and Meta-Analysis (PRISMA) (Figure 1). Available demographic, clinical, radiological, and histopathological data corresponding to individual patients were extracted from available reports using a standardized approach. For consistent and reliable reporting, patient and GAG characteristics were collected only when specified for a particular GAG and/or within a specified individual. The following patient and GAG characteristics were collected: person’s age, gender, comorbidities and/or past medical history, family history, number of GAGs, size(s) of GAG(s), location(s) of GAG(s), imaging features of GAG(s) (including imaging modality used), presenting sign(s) and symptom(s), ameliorating and/or exacerbating factors, treatments including medications, surgeries, interventions, and outcome. GAG dimension (i.e., maximum length or diameter) was recorded only when documented on cross-section (in mm or cm) for an individual GAG in a specific patient, whereas area and volumetric GAG size measurements or indiscriminate size ranges within persons, or within case series, were not included. Summary measures with age and sex are reported as descriptive statistics.

## 3. Results

### 3.1. Reports and GAG Cases

The published literature from 1973 to 2023 yielded 41 publications describing GAGs in 169 persons [3,7,8,9,10,11,12,13,14,15,16,17,18,19,20,21,22,23,24,25,26,27,28,29,30,31,32,33,34,35,36,37,38,39,40,41,42,43,44,45,46] (Table S1). This incorporated reports of 146 persons each with a single GAG; 21 persons with at least two GAGs [7,8,9,10,11]; and 2 persons who each exhibited “multiple” GAGs, with three [12] or four [8] GAGs being discernible on presented imaging. Considering the above, the review yielded a total of at least 195 reported GAGs. Of these, 164 (84%) were intrasinus-type, 30 (15%) were calvarial-type, and 1 (1%) was mixed-type; the specific location was recorded in 182 (93%); clinical history was available for 92 (47%); imaging data were available in 140 (72%); and histologic data were available in 52 (27%) [3,9,10,13,14,15,16]. Sex was available for 147 of 169 persons (87%) [3,10,13,14,15,16]. Among the GAG cases with histology, 14 presented at surgery [3,12,13,14,15,16,45], and 27 presented at autopsy [9,10]. Patients with GAGs exhibited variable demographic and GAG characteristics, as summarized in Table 1 and Table 2.

### 3.2. Demographic Features

**Figure 2:**
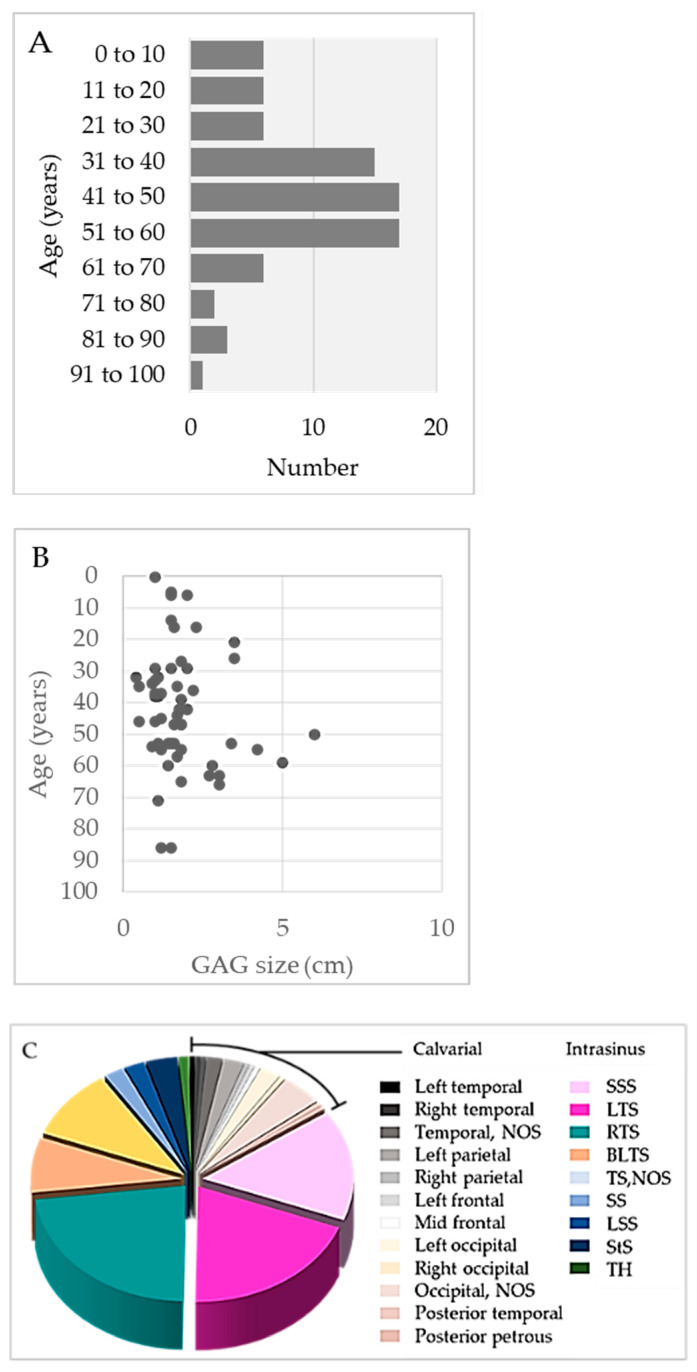
(**A**) Summary of the age distribution of persons with GAGs, according to decade of life. (**B**) GAG size distribution according to age. (**C**) Summary of GAG distribution by location.

Of the 147 persons with documented gender, GAGs involved 80 (54%) females and 67 (46%) males and were similarly distributed across sex (1.2:1 female-to-male ratio) (Table 2). The afflicted persons included infants [15], children [10,16,17,18,19,20], adolescents [17,21,22,23,24], and adults across a wide age spectrum [10,15,45] (range, 0.33 to 91 years; mean, 43 ± 20 years). Interestingly, one pediatric case was reported by parents since birth [16]. The mean number of GAGs per person was 1.0 and the overall number of GAGs recorded per decade of life is depicted in Figure 2A. Most persons with GAGs exhibited no comorbidities or past medical history (Table 1) but 4 of 169 persons (2%) were noted to be moderately obese or had cerebral small vessel disease [7,25,26]; 1 of 169 persons (1%) had a history of retrobulbar neuritis [3]; and 1 of 169 persons (1%) had staring episodes as well as a family history of seizures [10].

### 3.3. GAG Size and Morphology

The size distribution of GAGs is summarized in Figure 2B. The mean diameter of GAGs was 1.9 cm ± 1.1 cm (standard deviation), though diameters ranged from 0.4 to 6.0 cm (Table 2). Notably, five reported GAGs measured less than 1 cm in diameter (range: 4 to 9 mm) [26,32,33]. There was no direct correlation of GAG size with age [11]. The person’s age and GAG size were recorded for only 24 persons with recorded gender. Analyses among these cases revealed no statistically significant difference in mean age across male versus female individuals (females, 38 years; males, 45 years). However, a statistically significant difference was noted for GAG diameter across sex (females, 1.78 cm; males, 3.39 cm; *p* < 0.05) [3,13,15,16,20,23,26,27,28,29,30,31,32,33]. GAGs also varied in shape. Typically, GAGs were well-defined, nodular, round-to-ovoid structures, but others were irregular and a discrete, oblong vermiform shape was also characterized in one person (1%) [31].

### 3.4. Anatomical Distribution and Frequency

The majority of GAGs presented along SSS or transverse sinuses, or in parasagittal brain regions, whereas a subset involved the temporal bone (Figure 2C) and caused the compression of inner or middle ear structures [14,37]. Postmortem DVS studies reveal GAGs in 3.68–20% of adolescent and adult autopsies [9,10]. However, these analyses likely underestimate the true number of GAGs since they did not examine calvarial-type GAGs. Although imaging series are on record [11,18], no imaging study has analyzed the true prevalence of GAGs in live persons.

### 3.5. Reported GAG Histology

**Figure 3:**
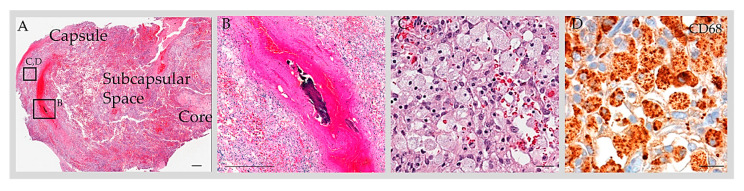
Anatomy of GAG. H&E-stained section of a GAG dome that was resected from an adult patient with post-traumatic headache revealed a multilaminar structure composed of a capsule, subcapsular space, and core (**A**). The subcapsular space contained blood and cells. On a high-power exam, a thrombosed vein (**B**) and foam cells (**C**) were present within the structure. Immunohistochemistry for CD68 highlighted a prominent number of cells, consistent with macrophages (**D**). Scale bars = (**A**,**B**), 100 µm; (**C**,**D**), 10 µm. Copped areas (black boxes in (**A**)) are shown in (**B**–**D**). Images reproduced from *Int. J. Mol. Sci.* **2023**, *24*, 11410 [45].

Fourteen calvarial-type or mixed-type GAGs were evaluated histologically [3,13,14,15,16,45] (Table S1). Surgically resected bone and soft tissue elements from these structures were studied with routine hematoxylin and eosin (H&E) staining following the decalcification of bone tissues and revealed collagen and meningothelium. The workup of two cases incorporated immunohistochemistry analysis [13,45]. Both of these cases were analyzed with the use of the anti-EMA label, which confirmed the meningothelial component [13,45]. One case that underwent comprehensive immunohistochemistry workup additionally revealed the presence of S100-positive nerve twigs; CD68-positive, CD138-positive, or CD45-positive immune cells (consistent with the presence of foam cells or monocytes/macrophages, plasma cells, or lymphocytes, respectively) (Figure 3); and CD31-positive, CD34-positive, and D2-40-positive capillary vessels within the GAG (consistent with blood capillary vessels and/or lymphatic capillary vessels). A thrombosed vein, hemorrhage, lymphatic vascular obliteration, and meningothelial hyperplasia were also present within this reactive GAG (Figure 3) [45]. Histology on 8 of 14 (57%) GAGs confirmed diploic space infiltration by GAGs [3,13,14,15,16]. In 5 of 14 (36%) cases, a large CSF-filled central cavity was reported rather than a dense collagen core, and these were therefore reported to mimic unilocular cysts [3,13,15]. In 14 of 14 (100%) cases, the outer GAG surfaces were covered by arachnoid or dural cells, rather than by endothelium [3,13,14,15,16]. On a retrospective review of published histological images, 3 of 14 (21%) cases demonstrate apparent mononuclear immune cell infiltrate within the GAG core, though this was not characterized as immune cells in the original reports [14,15]. Moreover, 2 of 14 (14%) described the presence of fat cells, though a retrospective review of published histology images suggests that these were instead foam cells (i.e., lipid-laden monocytes/macrophages) that had been misinterpreted on histologic assessment [16].

At least 27 DVS-type GAGs were identified on postmortem DVS examination [9,10,15] (Table S1). However, their tissue composites were not analyzed in detail. The largest population-based anatomical study of DVS-type GAGs consisted of a postmortem investigation published by Haybaeck et al. [9] and incorporated data from H&E and Elastica van Gieson stains as well as immunohistochemistry preparations incorporating labels for vimentin, desmin, EMA, and S100. In this series, intrasinus GAGs were reported to consist of dense collagen and meningothelial cell clusters covered by an endothelial cell layer. Mamourian et al. [10] describe large, centrally-placed blood vessels within three DVS-type GAGs from two patients although characterization of the tissue component was limited.

### 3.6. Signs and Symptoms

While some reports define GAGs as normal AG variants of no known clinical significance [10,34], heterogeneous acute, subacute, and/or chronic signs and symptoms have been reported in association with many GAG cases (Table 3). The most common presenting signs in persons with GAGs included headache (32 of 169 persons, 19%), vision change (10 of 169 persons, 6%), hearing change (9 of 169 persons, 5%), vertigo (6 of 169 persons, 4%), papilledema, and intracranial hypertension (each in 4 of 169 persons, 2%). Interestingly, 1 of 169 patients (1%) presented with a so-called laughing headache [7]. More ominous symptoms such as a change in consciousness, loss of consciousness, or seizure (each involving 2%) and meningism, neck pain, fever, and facial droop (each involving 1%) were also noted. Interestingly, 1 of 169 patients (1%) presented with repetitive hemorrhagic episodes, and 38 of 169 patients (22%) exhibited the herniation of brain parenchyma into a calvarial-type or DVS-type GAG, with involvement of cerebral cortical and/or cerebellar foliar tissue (Table 2). A significant proportion of patients with herniated brain tissue, including one 5-year-old child, exhibited evidence of brain injury (Table 2) [8,17,18,20,38]. In a series of 27 patients, Gozgec et al. [17] reported a statistically significant positive correlation between the frequency of herniated brain damage and GAG size (*p* < 0.05).

Some afflicted persons indicated that symptoms had been ongoing for several years, or for decades prior to diagnosis [26,29,45]. Acute clinical events that exacerbated GAG symptoms were present in 9 of 169 persons (5%) and a relieving factor, i.e., internal jugular venous compression that mitigated pulsatile tinnitus, was noted in 6 of 169 persons (4%) (Table 2; Table S1). A total of 3 of 169 (2%) patients with auditory changes complained of pulsatile tinnitus with “whooshing”, “swooshing”, or “sloshing” sounds [26,28,29]. All of these patients had transverse sinus or posterior temporal bone involvement by GAG [26,29], and one patient indicated that the frequency of the perceived auditory change was constant with her heartbeat [29]. Several patients indicated that GAG-associated symptoms had a significant impact on their quality of life or interfered with activities of daily living [7,17,26,29,45].

### 3.7. Imaging Features

**Figure 4:**
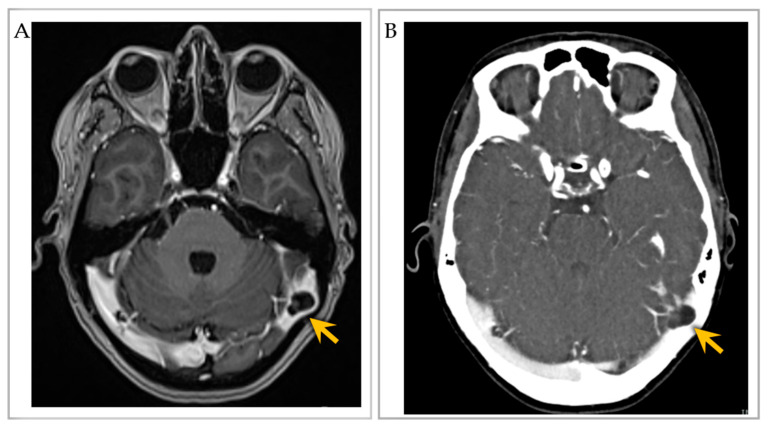
Imaging of an individual with multiple intrasinus-type GAGs. (**A**) Post-contrast T1-weighted brain MRI axial image shows two GAGs along the right and left lateral transverse sinuses, with severe sinus narrowing on the left (arrow). (**B**) CT venogram axial image further depicts the two GAGs with severe left lateral transverse sinus luminal narrowing (arrow). Images reproduced from *Int. J. Mol. Sci.* **2023**, *24*, 11410 [45].

Diagnosis of GAG on imaging workup (Table S1) was accomplished by visualization of round-to-ovoid, irregular or oblong, unilocular or multilocular cystic-appearing structures with or without internal septations, and with internal CSF-like density or signal intensity and communication with the subarachnoid space on MRI with and/or without contrast [8,13,15,17,19,20,22,23,24,25,26,28,30,31,36,37,38,39] (Figure 4A), MR angiogram/venogram [10,23,25,34,35,36,39,40,41,42], CT with and/or without contrast, or CT angiogram [8,21,25,27,28,31,34,35] (Figure 4B). GAGs were also identified as well-delineated focal calvarial defects on plain X-ray [13,15] or as focal filling defects within the DVS on conventional angiography and/or on cross-sectional studies [11]. GAGs with bone involvement caused smooth, evenly marginated impressions on the inner table of the skull and sometimes expanded into the diploic space, rarely eroding the outer skull table. Eight extrasinus-type GAGs that exhibited large “erosive” or “destructive” osteolytic calvarial defects were suspected to be malignant tumors [3,12,13,15,16]. MRI was the test of choice for differentiating GAGs from DVS thrombosis.

While the internal GAG characteristics generally paralleled those of CSF on CT and MRI, GAGs more commonly demonstrated internal vascular (i.e., presumed veins) and/or soft tissue elements that were not easily observable in smaller AGs. In an imaging review, brain parenchymal herniation into GAGs was found in 22% of DVS-type GAGs [18]. The internal MRI signal was CSF-incongruent in a majority of GAG cases [11,18] and this differential signal was most commonly identifiable on high-resolution T2-weighted or T2-FLAIR sequences [18]. In a retrospective MRI analysis of DVS-type GAGs published by Ogul et al. [18], vessels were identified in 33 of 45 GAGs (73.3%) and were best observable by contrast-enhanced dynamic MR venography or post-contrast high-resolution T1-weighted MPRAGE sequences. An internal GAG vein was demonstrated in 22 out of 26 (84.6%) female patients by dynamic MR venography and was significantly more common than in males (*p* = 0.04), although the reason for this sex difference is unclear [18].

### 3.8. Clinical Course and Medical Treatment

Several individuals experienced misdiagnosis or delayed diagnosis of GAG [7,22,43,45]. Since symptoms were sometimes initially interpreted as subacute or chronic DVS thrombosis, hypercoagulability workup and medical treatment were initiated to manage symptoms of presumed coagulopathy, infection, or related processes [12,14,19,22,23,25,26,33,35,36,39,41,43]. Patients were treated with acetazolamide [19,32,41,43], NSAID, anticephalgic medication, or other analgesia [12,23,33,34,43], other anticoagulants [22,35,36], antiepileptics [39], furosemide [41], mannitol [41], prednisolone [25], decongestants [14], and/or antibiotics [14,44] (each in 1 to 4 of 169 patients, or <3%). One patient with benign intracranial venous hypertension experienced symptomatic improvement following medical therapy with a month-long course of acetazolamide (2 g/day) and a 10-day course of prednisolone (100 mg/day) [25]. Interestingly, another patient developed a low-grade fever and underwent a broad medical workup with blood count, C-reactive protein, serum creatinine and blood urea nitrogen, glucose, electrolytes, liver enzymes, and lumbar spinal tap with Gram stain, which were all unremarkable [27]. A subset of patients responded to supportive treatment including intravenous fluids, analgesics, and rest [27]. Although follow-up imaging was not reported in many patients, radiological features were unchanged in the few persons who had been followed and medically managed for 4 to 24 months [10,45]. In one patient, GAG symptoms resolved spontaneously five days following depletive lumbar puncture, although clinical details and follow-up on this patient are sparse [24].

### 3.9. Interventional Surgical Treatment

Endovascular stenting was performed in seven patients with DVS-type GAGs who failed medical treatment and were found to have DVS stenosis [26,29,41,42] (Table S1). Seven of seven (100%) reported symptomatic improvement following the procedure. Zheng et al. [41] described a 34-year-old woman with a left transverse sinus GAG that was associated with headache, elevated venous pressure (proximal to the GAG), papilledema, and additional vision changes [41]. Her symptoms were refractory to acetazolamide, furosemide, and mannitol, yet transverse sinus stenting relieved her symptoms [41]. Similarly, Yang et al. [42], Gadot et al. [26], and Pereira et al. [29] described successful DVS stenting in middle-aged persons who had pulsatile tinnitus and transverse sinus GAGs with narrowing [41], with patients reporting immediate relief post-DVS stenting. For intervention, the Wallstent (Boston Scientific, Marlborough, MA, USA) was selected in two patients due to its closed-cell design and small free cell size that effectively excluded the GAG from the DVS lumen [29,42], whereas Cordis Precise Pro (Cardinal Health, Dublin, OH, USA) was placed in four patients [26] and Precise stent (Cordis Corporation, Miami Lakes, FL, USA) was used in one other [41]. Balloon angioplasty was unnecessary in all cases, as stenotic DVS regions expanded adequately following stent deployment [26,29,42]. No procedural complication was reported in any patients (0%). Following DVS stenting, patients were discharged on aspirin and clopidogrel (6-week course) [26], aspirin and clopidogrel (3-month course) [29], aspirin and ticagrelor (3-month course) [42], or warfarin followed by aspirin (3-month course of each) [41]. Seven of seven patients (100%) remained symptom-free at 3 months to 2.3 years post-stenting [26,29,41,42]. Demographic information was available for six of seven DVS-stented persons and included six women (100%) with a mean age of 48 ± 11 years.

### 3.10. Surgical Treatment

In total, 15 patients aged 4 months to 73 years underwent craniotomy with resection of DVS-type, calvarial-type, or mixed-type GAGs [3,13,14,15,16,28,32,45]. Procedures were undertaken in these patients for indications including facial droop, bone prominence, headache, weakness, sensory loss, expansile osteolytic lesion, and/or to prevent worsening of CSF leak or to exclude other pathologies (Table S1). These cases were centered at the parietal bone (four persons), temporal bone (three persons), frontal bone (two persons), occipital bone (five persons), or SSS (one person). Gacek et al. [14] reported a patient who failed decongestant, antibiotics, myringotomy, and tympanostomy tube placement, and eventually underwent surgical GAG resection in the temporal region in association with the repair of a CSF leak. Following surgery, the patient experienced an improvement in his symptoms [14].

At surgery, GAGs appeared as nodular projections or cysts that protruded through the dura, eroded into adjacent calvarium, and caused subcutaneous masses [15,45]. During resection, GAG structures appeared thin and/or unilocular or were composed of nodular collagenous structure that was present within scalloped bone [15]. The lesions occasionally exhibited a stalk [16,45], capsule [16,45], capsular vessels [16], or expressed CSF from its interior [16,45]. Significant hemorrhage was encountered upon resection of one GAG that exhibited capsular vessels but was controlled with bipolar coagulation [16]. GAG stalk amputation was performed in two patients [16], whereas the cranium and presumed GAG corpus (or body) were resected in six patients [3,15,45], whole GAG with stalk was resected in four patients [14], and the GAG apex was resected in one patient [45]. In order to control hemorrhage and/or CSF leakage following resection, Gelfoam (Upjohn Co., Kalamazoo, MI, USA) was applied to one GAG stalk margin that was located within the DVS [16] while a basal ligature was applied around the stalk of another GAG [45]. Titanium mesh cranioplasty was performed in two patients [45] whereas the skull defect was repaired with fascia and muscle in another individual [28]. Notably, two asymptomatic patients underwent a craniotomy to rule out alternate lesions, as indicated in dated reports [13,15]. Two other resected GAGs had been clinically mistaken for dermoid cysts [16]. One patient underwent initial biopsy resection due to a presumed cyst; however, after surgery, he complained of increasing occipital headache, localized tenderness, and swelling with a noted increase in mass size [32]. Therefore, a second surgery was undertaken on this patient eleven months after the first procedure [32]. At follow-up surgery, the GAG was coagulated, covered with fibrin foam, and a cranioplasty with acrylic resin was performed [32]. No other surgical complications werereported. Several patients reported improvement post-operatively without recurrence of symptoms, as documented two weeks to one year post-procedure [15,16,28,45].

## 4. Discussion

A literature review highlights the lack of consensus for a precise definition of the term GAG [11,22,23,35]. While some authors characterize AGs as “giant”-type when they exceed 1 cm in maximum dimension, others use this term for AGs that measure “approximately 10 mm in diameter” [9], or those that obliterate the DVS lumen or are associated with DVS dilatation, filling defect, and/or flow turbulence [34,35]. It should be noted, however, that GAG size parameters have not been correlated and/or discriminated between in vivo versus ex vivo investigations. Moreover, several enlarged GAGs, i.e., those measuring 1 cm or more in maximum dimension, were discovered incidentally and/or without associated DVS changes [34], whereas others were not reported as “giant”-type. Conversely, some subcentimeter “GAGs” were shown to be the cause of notable symptoms. Thus, the nomenclature and criteria for AG and GAG subclassification warrant further consideration [1,3,26]. In the future, “giant”-type arachnoid granulations may likely be reserved for reporting AGs meeting a defined size threshold of ≥1.0 cm, with “symptomatic”, “nonsymptomatic”, or “unclear symptoms” used as additional descriptive modifiers, independent of size.

It has been suggested that AGs and GAGs drain exclusively to the DVS and that their size is an indicator of CSF pressure [47]. But a review of published literature shows that while most reported GAGs are found in the vicinity of the DVS, subsets are remote from DVS locations. Upon review of published radiology data, it is noted that GAGs often exhibit internal vessels [18]. This finding was confirmed histologically in a recent radiological–pathological report on two specimens [45]. In one histologically well-characterized case, reactive changes present internally within an enlarged GAG also included post-traumatic venous thrombosis, hemorrhage, and inflammation with foam cell infiltrate that permeated, obliterated, and distended the subcapsular and central sinusoidal spaces [45]. Meningothelial hyperplasia, lymphatic vascular proliferation, and lymphatic vascular obliteration were also notable in this lesion [45], though these changes within the GAG interior were not previously observed in smaller, asymptomatic AGs harvested from human frontal regions [1]. These data therefore suggest that AG and GAG size differences may relate in part to structural and cellular variations [18,45].

While some reports define GAGs as incidental AG variants [10,34], many document heterogeneous acute, subacute, and/or chronic signs and symptoms in association with so-called GAGs, with some symptoms proven or strongly suspected to be due to AG or GAG lesions [26,45]. In certain patients, and due to limited information on this entity, GAGs may be misdiagnosed with alternate intracranial processes. Based on imaging and/or histologic appearances, the differential diagnosis of GAGs may include developmental aberrations (e.g., osteolysis, dysostosis, or meningoencephalocele), tumors or soft tissue masses (e.g., meningioma, hemangiomas, eosinophilic granuloma or Langerhans cell histiocytosis, glioma, myeloma, or metastases), cysts (e.g., epidermoid, arachnoid, dermoid, or epidermoid), reactive lesions (e.g., osteomyelitis), iatrogenic processes (e.g., burr hole and craniotomy defects), vascular channels (e.g., diploetic veins), or normal variants (e.g., calvarial venous lacunae). GAGs may also occasionally mimic meningothelial hyperplasia and may be misdiagnosed as a venous sinus pathology such as intravascular papillary endothelial hyperplasia (i.e., Masson’s tumor), other vascular neoplasms, or DVS thrombosis [17]. Middle ear GAGs may also mimic endolymphatic sac tumors or other inner ear lesions, whereas other differential diagnoses may include paraganglioma, chordoma, or chondromatous tumors, depending on the specific GAG location. GAG may also be an overlooked characteristic in a subset of persons with idiopathic intracranial hypertension syndrome [48].

## 5. Conclusions

While evidence pertaining to GAG anatomy and biology is limited, published data illustrate that these structures are variably symptomatic, may reach extraordinary dimensions (i.e., at least 6 cm in diameter [31]), exhibit heterogeneous anatomical and clinical characteristics, and are potentially treatable [15,45]. In some cases, GAGs may be numerous within a person, raise broad imaging and/or histopathological differential(s), and have the potential to cause grave symptoms. Available evidence consists primarily of case reports and rare retrospective case series that employ different terminologies and methodological approaches for GAG diagnosis and characterization. Limited follow up and the lack of comprehensive, systematic, and longitudinal reporting in prior patients hinders understanding of GAG etiologies and evolution. However, the literature demonstrates that GAGs are detectable in vivo by various imaging modalities. A recent analysis also depicts prominent vascular and immune changes within a histologically well-characterized specimen [45] and the present report consolidates demographic and clinical information from 195 cases reported to date. Systematic studies in large cohorts are needed to better elucidate the genesis, natural course, prognoses, and outcomes of symptomatic and/or enlarged AG subtypes in persons of variable age and with heterogeneous comorbid factors to better characterize thresholds for diagnosing and treating these variants by different modalities and in distinct brain locations.

## Figures and Tables

**Table 1:** Demographic information.

Age *	
Range *, years	0.33–91
Mean *, years (SD)	43 ± 20
Gender, n (%)	
Male	67/169 (40%)
Female	80/169 (47%)
Unknown	22/169 (13%)
Comorbidities/Past Medical History	
Obesity (low to moderate)	4/169 (2%)
Cerebral small vessel disease	4/169 (2%)
Systemic hypertension	3/169 (2%)
Nonspecific white matter change	2/169 (1%)
Intracranial hypertension	2/169 (1%)
Parkinson’s disease	2/169 (1%)
Carcinoma, colonic type	1/169 (1%)
Cerebral aneurysm	1/169 (1%)
Diabetes mellitus, type II	1/169 (1%)
Hypothyroidism	1/169 (1%)
Meningioma	1/169 (1%)
Retrobulbar neuritis	1/169 (1%)
Seizure disorder	1/169 (1%)
Tonsillectomy	1/169 (1%)
Number of GAG	
One GAG	149/169 (88%)
At least two GAG	18/169 (11%)
Evidence of more than two GAG	3/169 (2%)
Associated vascular abnormality	5/169 (3%)
Anterior cerebral artery aneurysm	1/169 (1%)
Arteriovenous fistula	1/169 (1%)
Benign intracranial venous hypertension	1/169 (1%)
High-riding jugular bulb	1/169 (1%)
Hypoplastic left jugular and left transverse veins	1/169 (1%)

Abbreviations: SD, standard deviation. Data are summarized for 169 persons or 195 GAGs. * Ages represent patient characteristics at the time of initial clinical presentation.

**Table 2:** Summary of GAG characteristics.

Size	
Range, cm	0.4–6.0
Mean, cm (SD)	1.9 ± 1.1
Location	
Intrasinus or DVS type	162/195 (83%)
Calvarial or diploic type	31/195 (16%)
Symptoms	
Absent (incidental)	9/169 (5%)
Present (symptomatic)	68/169 (40%)
Not Specified	93/169 (55%)
Onset/Exacerbating Factor	9/169 (5%)
With head position	2/169 (1%)
Head-down tilt Right-sided head turn	1/169 (1%) 1/169 (1%)
With acute event	8/169 (5%)
Acute head injury	3/169 (2%)
Acute exertion	3/169 (2%)
Acute heat exhaustion	1/169 (1%)
Laughing	1/169 (1%)
Sneezing	1/169 (1%)
Coughing	2/169 (1%)
With chronic event	2/169 (1%)
Remote head injury	3/169 (2%)
Relieving Factor	6/169 (4%)
I/L Jugular vein compression	6/169 (4%)
Myringotomy tube drainage	1/169 (1%)
Complications	38/169 (22%)
Intraocular peripapillary hemorrhage	1/169 (1%)
Brain herniation	38/169 (22%)
Cerebellum involvement	16/169 (9%)
Cerebrum involvement	13/169 (8%)
With brain atrophy	11/169 (7%)
With brain gliosis	6/169 (4%)
With brain infarction	1/169 (1%)

Abbreviations: cm, centimeter; I/L, ipsilateral; SD, standard deviation. Data are summarized for 169 persons or 195 GAGs.

**Table 3:** Acute, subacute, or chronic sign or symptom.

Headache	32/169 (19%)
Vision change	10/169 (6%)
Hearing Change	9/169 (5%)
Vertigo	6/169 (4%)
Intracranial hypertension	4/169 (2%)
Mental status change or change in consciousness	4/169 (2%)
Other, NOS *	4/169 (2%)
Papilledema	4/169 (2%)
Paresthesia	4/169 (2%)
Mass	3/169 (2%)
Seizure	3/169 (2%)
Syncope or loss of consciousness	3/169 (2%)
Anxiety	2/169 (1%)
Nausea	2/169 (1%)
Neck pain	2/169 (1%)
Facial droop	1/169 (1%)
Chronic ataxia	1/169 (1%)
Elevated opening CSF pressure	1/169 (1%)
Low-grade fever	1/169 (1%)
Meningism	1/169 (1%)
Meningitis	1/169 (1%)
Optic disc nasal effacement	1/169 (1%)
Otitis media	1/169 (1%)
Otitis media, serous	1/169 (1%)
Pain, NOS	1/169 (1%)
Repetitive hemorrhagic episodes, NOS	1/169 (1%)

Abbreviations: CSF, cerebrospinal fluid; NOS, not otherwise specified. * “Other, NOS” included suspected convulsion in at least one patient [17]. Data are summarized for 169 persons.

## Data Availability

Not applicable.

## Supplementary Materials

The following supporting information can be downloaded at: https://www.mdpi.com/article/10.3390/ijms241613014/s1.

## Abbreviations

AG	arachnoid granulation
CSF	cerebrospinal fluid
CT	computed tomography
DVS	dural venous sinus
GAG	giant arachnoid granulation
MRI	magnetic resonance imaging
SSS	superior sagittal sinus
TS	transverse sinus
