# Supplementary material for: Giant Arachnoid Granulations: A Systematic Literature Review
Source: Int J Mol Sci. 2023 Aug 21;24(16):13014. doi: 10.3390/ijms241613014 (PMC10455743; doi:10.3390/ijms241613014)
Supplement: Supplementary file 1 [file ijms-24-13014-s001.zip › ijms-2537888-supplementary.pdf]

Table S1. Summary of included literature.

Ref	First Author	No. Persons	Multiple GAG	No. GAG	Location	Autopsy/ Histology	Radiology	Medical Therapy	Stent	Surgery	Comment
[3]	Scully	1	No	1	Cranial	Yes	Yes	None	No	Yes	Symptom onset with trauma
[7]	Giraud	1	Yes	2	DVS	No	Yes	None	No	No	Symptom onset with laugh, sneeze, and cough
[8]	Khan	1	Yes	4	Cranial	No	Yes	None	No	No	Intra-GAG brain herniation; transient facial droop
[9]	Haybaeck	24	Yes	37	DVS	Yes	No	None	No	No	
[10]	Mamourian	5	Yes	6	DVS	Yes	Yes	None	No	No	Symptom onset with exertion
[11]	Trimble	17	Yes	19	Cranial or DVS	No	Yes	None	No	No	
[12]	Lu	1	Yes	3	Cranial	No	Yes	Anticephalgic	No	No	
[13]	Chan	1	No	1	Cranial	Yes	Yes	None	No	Yes	
[14]	Gacek	4	No	4	Cranial	Yes	Yes	Decongestant, Antibiotics	No	Yes	
[15]	Rosenberg	4	No	4	Cranial	Yes	Yes	None	No	Yes	Unilocular cysts with bone remodeling
[16]	Beatty	2	No	2	Cranial & DVS	Yes	Yes	None	No	Yes	Present since birth in one patient
[17]	Gozgec	27	No	27	Cranial or DVS	No	Yes	None	No	No	Intra-GAG brain herniation
[18]	Ogul	45	No	45	DVS	No	Yes	None	No	No	Intra-GAG brain herniation
[19]	Park	1	No	1	DVS	No	Yes	Acetazolamide	No	No	Symptom onset with head injury
[20]	Sade	1	No	1	DVS	No	Yes	None	No	No	Intra-GAG brain herniation
[21]	Chin	1	No	1	DVS	No	Yes	None	No	No	Symptom onset with head injury
[22]	De Keyzer	3	No	3	DVS	No	Yes	Anticoagulant	No	No	
[23]	Umeh	1	No	1	DVS	No	Yes	NSAIDS	No	No	
[24]	Taieb	1	Yes	2	DVS	No	Yes	None	No	No	Recurrent transient visual loss
[25]	Kiroglu	1	No	1	DVS	No	Yes	Acetazolamide, prednisolone	No	No	
[26]	Gadot	4	No	4	DVS	No	Yes	Anticoagulant	Yes	No	Symptom relief with IJV compression
[27]	Peters	1	No	1	DVS	No	Yes	None	No	No	Symptom onset with heat exhaustion
[28]	Deep	2	No	2	Cranial	No	Yes	None	No	Yes	History of remote head trauma
[29]	Pereira	1	Yes	2	DVS	No	Yes	None	Yes	No	Symptom exacerbated by exercise and head-down tilt; improved with IJV compression
[30]	Ayaz	1	No	1	DVS	No	Yes	None	No	No	Hypoplastic L. IJV and L. transverse vein
[31]	Mamaliga	1	No	1	DVS	No	Yes	None	No	No	Altered mental status of unclear etiology
[32]	Blaauw	1	No	1	Cranial	No	Yes	Acetazolamide	No	Yes	History of remote trauma
[33]	Deprez	1	No	1	DVS	No	Yes	Anticephalgic	No	No	
[34]	Kan	1	No	1	DVS	No	Yes	Anticephalgic	No	No	Lumbar puncture: clear CSF without pleocytosis
[35]	Choi	1	No	1	DVS	No	Yes	Anticoagulant	No	No	
[36]	Sunbulli	1	No	1	DVS	No	Yes	Anticoagulant	No	No	
[37]	Monté	1	No	1	Cranial	No	Yes	None	No	No	
[38]	Rodrigues	1	No	1	Cranial	No	Yes	None	No	No	Intra-GAG brain herniation; acute transient paresthesia
[39]	Karegowda	1	No	1	DVS	No	Yes	Antiepileptics	No	No	Associated with L. thrombosed AVF

[40]	Arjona	1	No	1	DVS	No	Yes	None	No	No	
[41]	Zheng	1	No	1	DVS	No	Yes	Acetazolamide, Furosemide, Mannitol	Yes	No	
[42]	Yang	1	No	1	DVS	No	Yes	Aspirin, Warfarin	Yes	No	Symptom relief with IJV compression
[43]	Rosenberg	1	No	1	DVS	No	Yes	Acetazolamide, Aspirin	No	No	Hypercoagulability workup
[44]	Esposito	1	No	1	Cranial	No	Yes	Antibiotics	No	No	
[45]	Mehta	3	Yes	5	Cranial	Yes	Yes	Topiramate	No	Yes	Symptom onset with head injury
[46]	Browder	At least 1	Unclear	At least 1	DVS	Yes	No	None	No	No	

Abbreviations: AVF, arteriovenous fistula; IJV, internal jugular vein; L, left; No., number; Ref, reference number.
